# Appearance of claudin-5^+^ leukocytes in the central nervous system during neuroinflammation: a novel role for endothelial-derived extracellular vesicles

**DOI:** 10.1186/s12974-016-0755-8

**Published:** 2016-11-16

**Authors:** Debayon Paul, Valentina Baena, Shujun Ge, Xi Jiang, Evan R. Jellison, Timothy Kiprono, Dritan Agalliu, Joel S. Pachter

**Affiliations:** 1 Blood-Brain Barrier Laboratory, University of Connecticut Health Center, 263 Farmington Ave., Farmington, CT 06070 USA; 2 Department of Cell Biology, University of Connecticut Health Center, 263 Farmington Ave., Farmington, CT 06070 USA; 3 Department of Neuroscience, University of Connecticut Health Center, 263 Farmington Ave., Farmington, CT 06070 USA; 4 Department of Immunology, University of Connecticut Health Center, 263 Farmington Ave., Farmington, CT 06070 USA; 5 Department of Pathology and Cell Biology, Columbia University School of Medicine, 630 W 168th St, New York, NY 10032 USA

**Keywords:** Leukocytes, Blood-brain barrier, Transendothelial migration, Extracellular vesicles, Exosomes, Microvesicles

## Abstract

**Background:**

The mechanism of leukocyte transendothelial migration (TEM) across the highly restrictive blood-brain barrier (BBB) remains enigmatic, with paracellular TEM thought to require leukocytes to somehow navigate the obstructive endothelial tight junctions (TJs). Transient interactions between TJ proteins on the respective leukocyte and endothelial surfaces have been proposed as one mechanism for TEM. Given the expanding role of extracellular vesicles (EVs) in intercellular communication, we investigated whether EVs derived from brain microvascular endothelial cells (BMEC) of the BBB may play a role in transferring a major TJ protein, claudin-5 (CLN-5), to leukocytes as a possible basis for such a mechanism during neuroinflammation.

**Methods:**

High-resolution 3D confocal imaging was used to highlight CLN-5 immunoreactivity in the central nervous system (CNS) and on leukocytes of mice with the neuroinflammatory condition experimental autoimmune encephalomyelitis (EAE). Both Western blotting of circulating leukocytes from wild-type mice and fluorescence imaging of leukocyte-associated eGFP-CLN-5 in the blood and CNS of endothelial-targeted, Tie-2-eGFP-CLN-5 transgenic mice were used to confirm the presence of CLN-5 protein on these cells. EVs were isolated from TNF-α-stimulated BMEC cultures and blood plasma of Tie-2-eGFP-CLN-5 mice with EAE and evaluated for CLN-5 protein by Western blotting and fluorescence-activated cell sorting (FACS), respectively. Confocal imaging and FACS were used to detect binding of endothelial-derived EVs from these two sources to leukocytes in vitro. Serial electron microscopy (serial EM) and 3D contour-based surface reconstruction were employed to view EV-like structures at the leukocyte:BBB interface in situ in inflamed CNS microvessels.

**Results:**

A subpopulation of leukocytes immunoreactive for CLN-5 on their surface was seen to infiltrate the CNS of mice with EAE and reside in close apposition to inflamed vessels. Confocal imaging of immunostained samples and Western blotting established the presence of CLN-5^+^ leukocytes in blood as well, implying these cells are present prior to TEM. Moreover, imaging of inflamed CNS vessels and the associated perivascular cell infiltrates from Tie-2-eGFP-CLN-5 mice with EAE revealed leukocytes bearing the eGFP label, further supporting the hypothesis CLN-5 is transferred from endothelial cells to circulating leukocytes in vivo. Western blotting of BMEC-derived EVs, corresponding in size to both exosomes and microvesicles, and FACS analysis of plasma-derived EVs from Tie-2-eGFP-CLN-5 mice with EAE validated expression of CLN-5 by EVs of endothelial origin. Confocal imaging and FACS further revealed both PKH-67-labeled EVs from cultured BMECs and eGFP-CLN-5^+^ EVs from plasma of Tie-2-eGFP-CLN-5 mice with EAE can bind to leukocytes. Lastly, serial EM and 3D contour-based surface reconstruction revealed a close association of EV-like structures between the marginating leukocytes and BMECs in situ during EAE.

**Conclusions:**

During neuroinflammation, CLN-5^+^ leukocytes appear in the CNS, and both CLN-5^+^ leukocytes and CLN-5^+^ EVs are detected in the blood. As endothelial cells transfer CLN-5^+^ to leukocytes in vivo, and EVs released from BMEC bind to leukocytes in vitro, EVs may serve as the vehicles to transfer CLN-5 protein at sites of leukocyte:endothelial contact along the BBB. This action may be a prelude to facilitate TEM through the formation of temporary TJ protein bridges between these two cell types.

**Electronic supplementary material:**

The online version of this article (doi:10.1186/s12974-016-0755-8) contains supplementary material, which is available to authorized users.

## Background

The blood-brain barrier (BBB) lies at the specialized microvasculature of the central nervous system (CNS). Consisting of a unique, continuous endothelium supported by a composite basement membrane, astrocyte end feet, and pericytes, its preeminent role is to strictly regulate the passage of soluble and cellular elements between the circulation and CNS by imposing severe restrictions on solute transport and cellular migration [1–4]. These restrictions are thought to be due, in significant part, to the presence of junctional complexes between endothelial cells [2, 5–7]. Leukocytes can nevertheless cross the BBB in significant number during neuroinflammation [8–10], though the mechanism(s) remains vague. One school of thought is that leukocytes somehow negotiate the series of endothelial tight junctions (TJs) and adherens junctions (AJs) that normally restrict the aqueous, inter-endothelial space [11–14]. In fact, a “zipper mechanism” has been proposed suggesting some leukocytes might engage in transendothelial migration (TEM) across particular vascular beds by temporarily replacing endothelial junctional contacts with homophilic and/or heterophilic interactions between corresponding leukocyte and endothelial junctional/adhesion proteins [15–18]. But the conventional view has held circulating leukocytes do not express much if any junctional proteins—at least in the absence of disease [19]—and so far, there has been no evidence of the zipper mechanism in the CNS. Even the suggestion that such a mechanism operates centrally would minimally require confirmation of CNS leukocytes bearing TJ and/or AJ proteins.

Recent findings, however, support the existence of circulating leukocytes harboring various junctional proteins and indicate expression of these proteins is related to neuroinflammation. Specifically, Mandel et al. [20] observed TJ proteins of the claudin (CLN) family—notably CLN-1 (CLN-1^+^) and CLN-5 (CLN-5^+^)—in peripheral blood leukocytes (PBLs) from both healthy individuals and those affected with the neuroinflammatory condition multiple sclerosis (MS). They further noted an increase in CLN-1 and CLN-5 protein expression by PBLs from MS patients experiencing disease relapse, compared to healthy controls or patients in remission. Moreover, effective treatment of relapsing disease with anti-inflammatory glucocorticoids was accompanied by decreased PBL expression of CLN-5. These findings thus suggest a positive correlation between leukocyte TJ protein expression and severity of neuroinflammatory disease. Numerous other studies documenting the presence of many TJ and AJ proteins on various leukocyte and other immune cell populations in vitro and in vivo [21–28] underscore detection of these cells is not an isolated phenomenon but, instead, a more frequent occurrence that may be related to immune activation and/or function.

While ectopic expression of these junctional proteins by leukocytes may be achieved by endogenous synthesis, this could also potentially occur by transfer from other cell types—notably endothelial cells, which are a particularly rich source of TJ and AJ proteins [2, 7, 29]. One route for such transfer might be via extracellular vesicles (EVs, previously known as “microparticles”)—nano-size, membrane-bound structures shed from numerous cells types—which mediate intercellular communication and can convey a broad spectrum of bioactive molecules (including protein, messenger RNA (mRNA), miRNA, and DNA) over long and short distances [30–32]. EVs constitute a heterogeneous family of vesicles and are classified according to their size and route of derivation. Exosomes are generally 40–100 nm in diameter and originate from multivesicular endosome fusion with the plasma membrane, while microvesicles are in the 100–1000-nm range and arise from exocytic budding of the plasma membrane [33, 34]. Significantly, EVs can vary in their cargo and cellular targets [35]. The prospect of EVs acting as vehicles to transfer junctional proteins to leukocytes is particularly alluring for several reasons. EVs from varied sources have been reported to contain several junctional proteins [36–40] and mechanical injury to cultured brain microvascular endothelial cells (BMEC) found to stimulate release of EVs harboring the TJ protein occludin [41]. Moreover, several reports reinforce the idea that varied leukocyte subtypes are physiological targets of endothelial EVs. In vivo analysis has shown elevated endothelial EV-leukocyte complexes in plasma from MS patients during disease exacerbation [42]. And binding of endothelial EVs, obtained from blood of patients with severe systemic inflammatory response syndrome, to neutrophils has been demonstrated in vitro [43], as has binding of EVs from BMEC cultures to both monocytes [44] and lymphocytes [45]. That inflammation in and outside the CNS [46–51], and adhesion of leukocytes [52], each triggers release of EVs from endothelial cells supports a juxtacrine mechanism for endothelial EVs to transfer junctional proteins to leukocytes at or near the BBB during neuroinflammatory disease.

To address the issues of whether leukocytes bearing TJ proteins enter the CNS, and the possible origin of these proteins, initial studies were performed to identify leukocytes harboring the TJ protein CLN-5—a determinant of the BBB [53]—in the blood and spinal cord of wild-type mice with experimental autoimmune encephalomyelitis (EAE), an animal model of MS [54–56]. EAE was then induced in transgenic mice with eGFP-CLN-5 targeted to endothelial cells to examine if endothelial cells transfer CLN-5 to leukocytes. To additionally investigate the potential for EVs to act as vehicles for CLN-5 transfer, EVs released from cultured BMEC were evaluated for the presence of CLN-5 and ability to bind to leukocytes. EVs isolated from the blood of Tie-2-eGFP-CLN-5 transgenic mice were similarly evaluated for leukocyte binding capacity. Finally, serial electron microscopy and 3D rendering was employed to view possible EVs at the leukocyte:BBB interface in situ.

## Methods

### Mice

Wild-type, female C57BL/6 mice, aged 8 to 10 weeks, were obtained from Charles River Laboratories, Inc. (Wilmington, MA). Transgenic C57BL/6J mice, expressing reporter eGFP fused to CLN-5 protein, under direction of the endothelial Tie-2/Tek-1 promoter/enhancer [57], referred to from hereon as Tie-2-eGFP-CLN-5 mice, were obtained from Dr. Dritan Agalliu (Department of Pathology, Columbia University). All animal experimental procedures were performed following the Animal Care and Use Guidelines of the University of Connecticut Health Center (Animal Welfare Assurance A3471-01) and approved under protocol 100346-1214.

### EAE induction

EAE was induced in mice by active immunization with MOG_35–55_ peptide (MEVGWYRSPFSRVVHLYRNGK), of murine origin (W. M. Keck Biotechnology Resource Center, Yale University, New Haven, CT) and disease scored as described [58, 59]. Specific time points of analysis are referred to as “D” followed by the number of days after disease induction (e.g., D9 is 9 days after EAE induction).

### Mouse BMEC cultures

BMEC cultures were established from two sources: primary-derived BMEC or an immortalized cell line. Primary-derived BMEC were obtained from eGFP-CLN-5 mice by immuno-bead selection as previously described [60] and maintained in Dulbecco’s modified Eagle’s medium F-12 (DMEM-F-12) containing 10% plasma-derived horse serum, 10% fetal bovine serum, 1% antibiotic-antimycotic (all from ThermoFisher Scientific, Grand Island, NY), 100 μg/ml heparin, and 100 μg/ml endothelial cell growth supplement (BD Biosciences, Bedford, MA), at 37 °C, 5% CO_2_. These were used to confirm both the classical junctional localization of eGFP-CLN-5 (Additional file 1: Figure S1) and release of eGFP-CLN-5^+^ EVs (data not shown) from freshly derived BMEC. It is thus assumed all eGFP signal associated with endothelial cells, leukocytes, and EVs stems from eGFP-labeled CLN-5. The immortalized cell line bEND3, derived from a mouse brain capillary hemangioma [61] and expressing CLN-5 [62], was obtained from the American Type Culture Collection and grown in DMEM containing 10% fetal bovine serum and 1% antibiotic-antimycotic, at 37 °C, 5% CO_2_. These cells were employed to obtain EVs in bulk for Western blotting analysis and leukocyte binding.

### Isolation of EVs (exosomes and microvesicles) from BMEC cultures

BMEC were cultured to confluence for all experiments. Prior to experimentation, cells were switched to media supplemented with exosome-depleted fetal bovine serum (Exo-FBS™; Systems Biosciences, Mountainview, CA) and grown for an additional 12 h with 10 ng/ml TNF-α to mimic the neuroinflammatory environment of EAE [63, 64] as well as stimulate EV release [44, 65]. EVs were then isolated from the BMEC supernatant and resolved into exosome and microvesicle subtypes by differential centrifugation using a combination of established protocols [39, 66]. The BMEC supernatant was sequentially spun at 300×*g* for 10 min at 4 °C, 2000×*g* for 10 min at 4 °C using a swing-bucket rotor and Eppendorf 5804R centrifuge (Eppendorf, Hauppauge, NY), and finally 8000×*g* for 30 min at 4 °C in a fixed-angle rotor and Sorvall RC-5C Plus centrifuge (Sorvall-Thermo Scientific, Dubuque, IA) to remove whole cells, large cell fragments, and apoptotic bodies, respectively. The clarified supernatant was then spun at 20,000×*g* for 30 min at 4 °C to pellet EVs of larger microvesicle-size and the resulting supernatant spun again at 60,000×*g* for 30 min at 4 °C to pellet smaller-size microvesicles and possible exosome aggregates using a Beckman TL-100 ultracentrifuge (Beckman Coulter, Indianapolis, IN). The post-60,000×*g* supernatant was then spun at 100,000×*g* for 60 min at 4 °C to pellet exosome-size EVs. Validation of the respective EV subtypes was performed using high-resolution particle-size profiling with a nanoparticle tracking analysis (NTA) device (NS300; Malvern Instruments, Westborough, MA) (Additional file 2: Figure S2). In some cases, “total” EVs containing both exosomes and microvesicles were isolated by centrifuging the clarified supernatant (generated after sequential 300×*g*, 2000×*g*, and 8000×*g* spins) directly at 100,000×*g* for 60 min.

### Co-isolation of leukocytes and EVs (exosomes and microvesicles) from blood

Both EVs and PBLs were isolated from the same population of mice with EAE at D8 post-induction, a time at which significant physical disruption of the BBB is not yet apparent [67]. A total of 10 mice were used for each preparation. Mice were anesthetized by intraperitoneal injection of ketamine (80 mg/kg) and xylazine (10 mg/kg) in phosphate-buffered saline (PBS). A 3 ml syringe with a 25 gauge needle was briefly flushed with 0.109 M (3.2%) sodium citrate (BD, Franklin Lakes, NJ), and blood was slowly acquired using transcardiac puncture. A total of 3 ml of fresh anticoagulant-treated blood was diluted 1:1 with Hank’s Balanced Salt Solution (HBSS; ThermoFisher Scientific, Grand Island, NY) at 25 °C by gently inverting the tube and slowly layered on 3 ml of Ficoll-Paque PLUS (Sigma-Aldrich, St. Louis, MO) in a 15 ml Falcon tube. Samples were spun at 400×*g* for 40 min at 25 °C in a swing-bucket centrifuge (Eppendorf 5804R) without acceleration or brakes.

The “buffy” coat layer from the Ficoll-Paque PLUS gradient was aspirated using a 1 ml pipette, washed in three volumes of HBSS, and spun at 400×*g* for 10 min. After discarding the supernatant, 1 ml of RBC lysis buffer was added to each tube to eliminate RBC contamination and incubated for 5 min at 25 °C. The sample was then neutralized with three volumes of HBSS and spun at 400×*g* for 10 min. The pellet was resuspended in HBSS and the gradient-purified leukocytes used for Western blot analysis and immunocytochemistry.

The upper layer from the Ficoll-Paque PLUS gradient, containing plasma, was used for EV (exosome and microvesicle) isolation. Plasma was subject to the same differential centrifugation protocol as performed on the BMEC supernatant.

### Western blotting

Isolated leukocytes and EVs were solubilized in 8 M urea containing protease inhibitor cocktail (Sigma-Aldrich, St. Louis, MO). Protein concentration was assayed by the Micro BCA protein assay kit (ThermoFisher Scientific, Grand Island, NY). Lysates containing 10–30 μg leukocyte protein, or 10 μg of EV protein, were separated by electrophoresis on 4–20% Mini-PROTEAN® TGX™ Precast SDS-PAGE gels and transferred onto PVDF membranes (Bio-Rad Laboratories, Hercules, CA). Membranes were then blocked with 5% bovine serum albumin (BSA) in Tris-buffered saline with Tween-20 (TBST) (ThermoFisher Scientific, Grand Island, NY) for 1 h at room temperature, followed by incubation overnight at 4 °C with the CLN-5 antibody (1:200; Life Technologies, Carlsbad, CA) diluted in 5% BSA in TBST. Following incubation with anti-mouse HRP-conjugated secondary antibody (1:400; Cell Signaling), blots were developed using the chemiluminescent HRP substrate kit (SuperSignal West Pico Chemiluminescent Substrate, ThermoFisher Scientific, Grand Island, NY) and signal detected using a G:Box XX6 digital gel imager (Syngene, Frederick, MD). Images were acquired by GeneSys software (Syngene, Frederick, MD).

### Quantitative RT-PCR

Total RNA was extracted from cells using the complementary DNA (cDNA) direct lysis buffer. cDNA was synthesized from the total RNA using a SuperScript III first-strand synthesis system for RT-PCR with a standard protocol. Measurements of cDNA levels were performed by quantitative RT-PCR. Relative claudin-5 gene expression values were expressed as percentage of RPL-19, as described previously [59].

### High-resolution 3D imaging of CLN-5^+^ leukocytes

D9 EAE was selected for immunohistological analysis of early leukocytic infiltrates into the CNS as this time point has been previously reported to display the earliest signs of focal increase in perivascular cellularity representing infiltrating leukocytes in spinal cord microvessels [67]. Tissue processing, immunofluorescent staining of 60-μm-thick cryosections, and high-resolution imaging of inflamed microvessels along with the associated perivascular infiltrates in confocal z-stack images were performed using a Zeiss LSM 510 Meta confocal microscope equipped with a 100x plan-apochromatic oil immersion lens, 1.4 NA as detailed previously [58, 67]. Alexa® 488-conjugated CLN-5 antibody (Clone 4C3C2, Life Technologies, Carlsbad, CA) was used for immunostaining. Isolated leukocytes were plated on poly-l-lysine-coated eight-well chamber slides (ThermoFisher Scientific, Grand Island, NY), immunostained, and imaged similarly to sections.

### Image analysis of EV:leukocyte binding

Exosome- and microvesicle-size EVs were separately labeled with green fluorescent membrane dye PKH-67 (Life Technologies, Carlsbad, CA). For binding studies, PBLs were isolated from naïve mice using an Acrodisc® white blood cell syringe filter (Pall Life Sciences, Port Washington, NY), as this procedure was faster than gradient purification and allowed for heightened viability. Freshly isolated PBL leukocytes were labeled with red fluorescent membrane dye, PKH-26 (Life Technologies). PKH-67-labeled EVs were added to PKH-26-labeled leukocytes in 500 μl microfuge tubes (ThermoFisher Scientific, Grand Island, NY) and mixed overnight in Ca^2+/^Mg^2+^-free HBSS supplemented with 2% exosome-depleted bovine serum (System Biosciences) at 4 °C using a 360° rotator (Barnstead/ThermoFisher Scientific). Following HBSS wash, the leukocyte-EV complexes were spun down at 400×*g* to remove any unbound EVs. The leukocyte-EV complexes were allowed to settle for an additional 2 h on poly-l-lysine-coated eight-well chamber slides (ThermoFisher Scientific, Grand Island, NY) for imaging. Adherent leukocyte-EV complexes were fixed in 2% paraformaldehyde in PBS. High-resolution images were acquired using 63x plan-neofluar oil immersion lens, 1.25 NA, as described above for 3D imaging of CLN-5^+^ leukocytes in spinal cord sections. All binding reactions were set up between leukocytes from naïve mice and EVs from mice at D8 EAE. The reasoning for this design was that activated leukocytes from mice with EAE might be nearly saturated with previously bound EVs in vivo and thus might display minimal binding in vitro, while EVs released during neuroinflammation might be more likely to express CLN-5.

### Bone marrow chimeras

Six-week-old C57BL/6J Tie-2-eGFP-CLN-5 transgenic mice (CD45.2) were lethally irradiated (1000 rad) to achieve myeloablation. Six hours later, hematopoietic reconstitution was performed by retro-orbital administration of 200 μl sterile PBS containing 1 × 10^7^ bone marrow cells derived from the femur and tibia of adult, wild-type (WT) congenic C57BL/6J mice (CD45.1/CD45.2). At approximately 9 weeks after bone marrow reconstitution, peripheral blood chimerism was assessed by fluorescence-activated cell sorting (FACS) analysis of tail blood samples.

### FACS analysis

#### Detection of CLN-5-immunostained PBLs

PBLs were isolated from WT mice blood at D8 post-EAE using Acrodisc® white blood cell syringe filter (Pall Life Sciences, Port Washington, NY). Following 4% PFA fixation, PBLs were either left unpermeabilized or permeabilized with 0.2% Triton X-100 for 30 min and incubated overnight at 4 °C with anti-CLN-5-Alexa® 488 antibody (1:150; Life Technologies, Carlsbad, CA). Unlabelled PBLs and PBLs incubated with Alexa® 488-conjugated isotype controls were used for compensation. The intensity of CLN-5 immunostaining in PBL suspensions with or without permeabilization was analyzed on a FACS LSR II (Becton Dickinson, Franklin Lakes, NJ). Data analysis was performed with FlowJo software version 9 (Treestar, Ashland, OR).

#### Detection of eGFP-CLN-5^+^ PBLs

PBLs were obtained from blood of Tie-2-eGFP-CLN-5 mice with EAE and the FACS analysis performed in a manner similar to that described for CLN-5 immunostaining.

#### Analysis of peripheral PBL chimerism

Efficient reconstitution of the chimeric mice was confirmed by determining percentage donor CD45.1^+^ CD45.2^+^ cells in the blood of irradiated Tie-2-eGFP-CLN-5 mice. Approximately 100 μl blood was collected from anesthetized mice via tail bleed and RBC contamination eliminated using RBC lysis buffer. Following neutralization of the sample with HBSS, PBLs were obtained by centrifugation, 400×*g* for 10 min. PBLs were resuspended in PBS containing 2% FCS at 4 °C and stained with anti-CD45.1-FITC, anti-CD45.2-APC (both from Tonbo Biosciences, San Diego, CA), and anti-CD3-eF450 (eBiosciences, San Diego, CA). Cell suspensions were analyzed by FACS as described for CLN-5-immunostained and eGFP-CLN-5^+^ PBLs.

#### FACS sorting eGFP-CLN-5^+^ EVs and FACS analysis of eGFP-CLN-5^+^ EV:PBL binding

A FACSAria™ II (BD Biosciences, San Jose, CA) was used to sort eGFP-CLN-5^+^ EVs from the BMEC culture supernatant or blood plasma of Tie-2-eGFP-CLN-5 transgenic mice. A 130 μm nozzle and low 10 PSI sheath pressure were used while running at the slowest sample speed, so as to reduce the size of the sample core stream while maximizing laser excitation of small particles. Nano-fluorescent beads (Spherotech, Lake Forest, IL) were used to determine the limit of detection of submicron particles using both side scatter and green fluorescence detectors and to gate on events that lay outside of “noise.” For binding analysis, EVs alone (either isolated from the Tie-2-eGFP-CLN-5 mice or PKH-67-labeled EVs isolated from WT mice with EAE) and PKH-26-labeled PBLs alone were used to compensate for the respective unbound populations. Leukocytes were gated to omit debris events based on forward and side light scatter. Single leukocytes were defined as having one forward scatter pulse width per forward scatter trigger (Additional file 3: Figure S3). Percentage(s) of double-positive events was considered as a relative measure of EV:PBL binding.

### Serial electron microscopy (serial EM)

Serial EM was performed as previously detailed [68, 69]. Mice were anesthetized with ketamine/xylazine and transcardially perfused first with PBS to wash off the blood and then with 2.5% glutaraldehyde and 2.0% paraformaldehyde in 0.1 M cacodylate buffer through the left ventricle of the heart. The spinal cord was isolated by laminectomy and fixed for an additional 3–4 h in the same fixative and then rinsed and stored in 0.1 M cacodylate buffer at 4 °C until further processing. The lumbar section of the spinal cord was cut with a razor blade into ~1 mm-thick slices and rinsed in 0.1 M cacodylate buffer several times. The samples were processed using the ROTO protocol [70] and then dehydrated in graded ethanol solutions and embedded in epoxy resin (Polybed Polysciences, Warrington, PA).

After the samples were polymerized, the face of each block was shaped to a ~2 × 3 mm rectangle using a diamond trimming knife. Thin sections, 60 nm thick, were cut using a microtome (Leica EM UC7, Buffalo Grove, IL) and a diamond knife. Serial sections were collected on Kapton tape (glow-discharged to minimize wrinkling of sections) using the ATUM tape collector [71]. The tape with sections was cut into strips and mounted on 4 in. silicon wafers (University Wafers, South Boston, MA) and then carbon-coated for electron grounding (Denton 502B, Moorestown, NJ).

Sections were imaged using a field emission scanning EM (Zeiss Sigma FE-SEM; Peabody, MA) in backscatter mode (10 keV electrons, ~5 nA beam current). A high-precision map of the sections on the wafer (±4 μm) was generated, and then, the Atlas Large Area Imaging software (Fibics Inc., Ottawa, Ontario, Canada) was used to automatically image a ~65 μm × 65 μm field of the serial sections at 5–7 nm/pixel resolution (12,288 × 12,288 pixels). The images were aligned using the Linear Alignment with SIFT algorithm (FIJI, ImageJ) and reconstructed using Imaris®.

### 3D contour surface creation of serial EM slices using Imaris

To visualize the endothelium and possible EVs at the site of leukocyte adhesion in 3D, the serial EM slices were first imported into Imaris for volume rendering. Manual contour tracing was then performed by cursoring out the endothelium, EV-like structures and the adherent leukocyte(s) of interest in each serial EM z-slice and the individual contours merged into a 3D contour surface, as detailed previously [58, 72].

## Results

### Presence of CLN-5^+^ leukocytes within the CNS during EAE

**Fig. 1:**
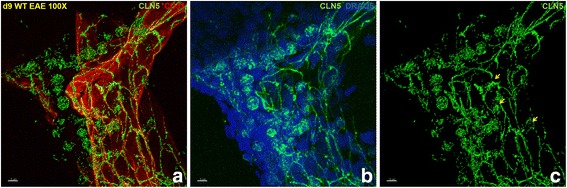
CLN-5^+^ leukocytes are present in the CNS during early EAE. z-stack confocal images acquired from the same spinal cord cryosection of a WT mouse at D9 EAE are shown. **a** Immunostaining of TJ protein CLN-5 (*green*) and CD31 (*red*) to identify endothelial cells, showing CLN-5 is present on both leukocytes and at endothelial junctions. **b** DRAQ5 staining (*blue*) highlights the cellularity associated with CNS-infiltrating leukocytes, revealing CLN-5^+^ leukocytes (*green*) comprise a subset of invading cells. **c** Immunostaining of TJ protein CLN-5 only; *arrows* (*yellow*) indicate some CLN-5^+^ leukocytes are associated with areas of discontinuity of CLN-5 junctional staining. Leukocyte-associated CLN-5 immunoreactivity displays a punctate appearance


To view CLN-5^+^ leukocytes in the CNS during neuroinflammation, spinal cord sections of WT mice at D9 post-EAE induction—during the pre-clinical phase of disease—were immunostained and analyzed by high-resolution 3D fluorescence microscopy. This time point was specifically selected in case CLN-5^+^ leukocytes operate early in the extravasation process that drives pathogenesis—before substantial BBB disruption. Immunostaining of microvessels revealed the typical “chicken wire” pattern of CLN-5 along inter-endothelial cell borders [58, 67]. Additionally, leukocytes labeled with anti-CLN-5 were obvious within perivascular infiltrates surrounding inflamed vessels (Fig. 1; Additional file 4: Video 1) and revealed punctate immunostaining patterns that seemingly covered the cells. These CLN-5^+^ leukocytes were mostly visible along the meningeal microvessels and the infiltrating parenchymal vessels at this early time point in EAE (Additional file 5: Figure S4). CLN-5^+^ leukocytes also appeared to concentrate in regions proximal to focal discontinuities in vascular CLN-5 immunoreactivity that might reflect subtle changes in BBB integrity. Despite their obvious detection, CLN-5^+^ leukocytes constituted only a fraction of the invading leukocyte population. This laboratory previously found no evidence of CLN-5^+^ leukocytes—or any perivascular infiltrates—in spinal cord microvessels from naïve mice during an exhaustive analysis of CLN-5 distribution using the same identical staining and image acquisition parameters described in this report [58]. Appearance of CLN-5^+^ leukocytes thus accompanies early infiltrates during disease.

Additional file 4Video 1. 3D perspective of CLN-5^+^ leukocytes in the CNS during early EAE. 3D reconstruction of z-stack confocal images shown in Fig. 1 reveals the 3D distribution of extravasated CLN-5^+^ (green) leukocytes along an inflamed microvessel highlighted with CD31 (red) staining. DRAQ5 staining (blue) highlights the perivascular cellularity, showing CLN-5^+^ leukocytes comprise only a fraction of total CNS-infiltrating leukocytes.


### Detection of circulating CLN-5^+^ leukocytes (PBLs) during EAE

**Fig. 2:**
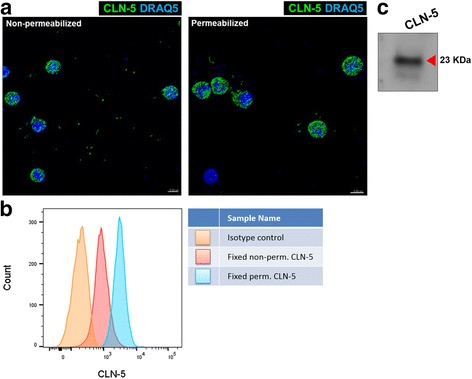
CLN-5^+^ leukocytes in circulation during early EAE. **a** Representative z-stack confocal images of PBLs isolated from WT mice at D8 EAE and immunostained with CLN-5 (*green isosurface*) under non-permeabilized or permeabilized (with Triton X-100) conditions. Leukocytes were obtained a day earlier than in Fig. 1, to ensure those CLN-5^+^ cells in the circulation had not yet all extravasated. **b** FACS analysis of leukocytes from D8 EAE mice immunostained with the same antibody shows CLN-5 staining under both non-permeabilized and permeabilized conditions. Leukocytes stained with an isotype antibody were used as control. **c** Western blot analysis of lysates from the same batch of leukocytes and the same antibody clone used in **a** and **b** showing a 23 kDa molecular weight band, consistent with the molecular weight of CLN-5

Next, the presence of CLN-5^+^ leukocytes in the circulation was evaluated. A similar, punctate, CLN-5 immunostaining pattern was seen on PBLs from mice with EAE as was observed on leukocytes in the CNS (Fig. 2a). This pattern was further observed in both detergent-permeabilized and non-permeabilized cells, consistent with the interpretation that the immunoreactive CLN-5 epitope is at least partially present on the cell surface. FACS analysis corroborated the imaging results, additionally revealing higher staining intensity in the permeabilized samples, wherein antibody may also access intracellular CLN-5 (Fig. 2b). To further assure these CLN-5^+^ cells were leukocytes and not circulating endothelial cells, PBLs were double-stained for CLN-5 and common leukocyte antigen, CD45 (Additional file 6: Figure S5). Though co-localization of CLN-5 and CD45 immunostaining was obvious in many cases, cells that brightly stained for CLN-5 showed a reduced intensity for CD45. This could possibly be due to steric hinderance between the antibodies, as double staining was best observed when cells were incubated with both antibodies simultaneously rather than sequentially (data not shown). Western blotting confirmed the expression of CLN-5 protein at the expected apparent Mw_r_ of approximately 23 kDa (Fig. 2c).

In addition to displaying CLN-5 protein, PBLs also demonstrated CLN-5 gene expression (Additional file 7: Figure S6). CLN-5 expression was detected even in PBLs from naïve mice but was significantly upregulated by D8 EAE and then decreased by D13.

### Endothelial origin of CLN-5 on leukocytes

**Fig. 3:**
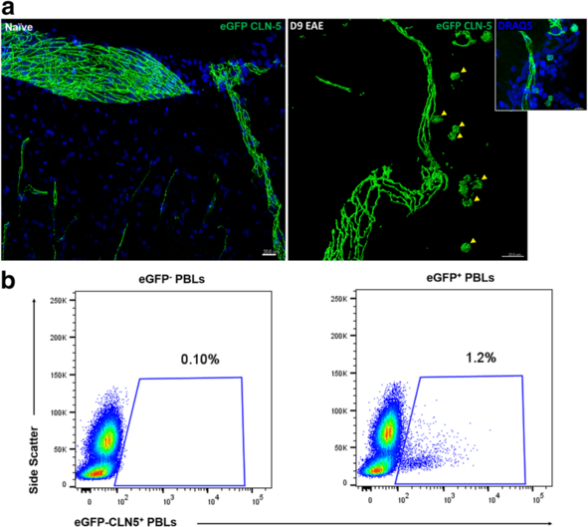
Appearance of eGFP-CLN-5^+^ leukocytes in Tie-2-eGFP-CLN-5 mice during EAE. **a** z-stack confocal images acquired from spinal cord cryosections from Tie-2-eGFP-CLN-5 mice showing distribution of eGFP-CLN-5 (*green*) in naïve venules and associated with an inflamed venule and perivascular leukocytes at D9 EAE. *Inset* shows DRAQ staining (*blue*) highlighting the extent of perivascular cellularity, representing leukocyte infiltrates and a minority fraction of eGFP-CLN-5^+^ leukocytes. The presence of eGFP-CLN-5^+^ leukocytes in the CNS is consistent with these cells having acquired eGFP-CLN-5 from endothelial sources. **b** PBLs were isolated from Tie-2-eGFP-CLN-5 mice (*n* = 5) at D8 post-EAE induction and then subjected to FACS analysis and percentage eGFP-CLN-5^+^ CD45^+^ recorded (*right*). Control PBLs from naïve, WT mice were used to set the gate (*left*)

Because expression of TJ proteins is typically reserved for endothelial and epithelial cells, the appearance of these proteins on leukocytes is regarded as ectopic [19]. Accordingly, the next experiments addressed whether endothelial cells—with which leukocytes come in intimate contact during inflammation—might be the likely origin of leukocyte-associated CLN-5. To verify endothelial origin of CLN-5 on leukocytes, Tie-2-eGFP-CLN-5 mice that demonstrate targeted expression of eGFP-CLN-5 in all endothelial cells [57] were used. First, localization of the eGFP signal at endothelial cell-cell junctions was confirmed in BMEC cultures (Additional file 1: Figure S1) and spinal cord cryosections from naïve Tie-2-eGFP-CLN-5 mice (Fig. 3a). The eGFP signal demonstrated an identical chicken wire distribution with anti-CLN-5 staining, underscoring both staining patterns reflect TJ-associated CLN-5. No infiltrating eGFP-CLN-5^+^ cells or other cellular infiltrates were seen in the perivascular spaces surrounding penetrating or parenchymal venules in naïve mice, consistent with the prior report from this laboratory documenting absence of immunostained CLN-5^+^ leukocytes and infiltrates from healthy CNS tissue [58]. Next, the appearance of eGFP-CLN-5^+^ leukocytes in the CNS was evaluated in Tie-2-eGFP-CLN-5 mice with EAE. Figure 3a shows eGFP-CLN-5^+^ leukocytes in the perivascular locale within the CNS, alongside an eGFP-CLN-5^+^ microvessel. Like that observed with anti-CLN-5 staining, the eGFP signal on leukocytes had a punctate display and appeared to cover the cells. Additionally, eGFP-CLN-5^+^ leukocytes—as for anti-CLN-5-stained leukocytes—represented only a fraction of the perivascular infiltrate and were absent from the CNS of naïve, Tie-2-eGFP-CLN-5 mice. This was in accord with a low fraction of eGFP-CLN-5^+^ PBLs (1.2%) in the blood of Tie-2-eGFP-CLN-5 mice at D8 post-EAE (Fig. 3b).

**Fig. 4:**
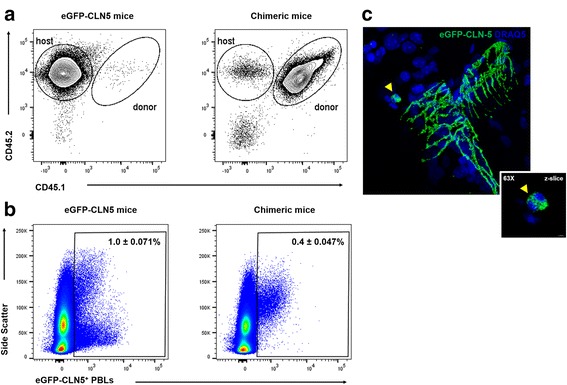
WT/Tie-2-eGFP-CLN-5 chimeras highlight endothelial origin of leukocyte CLN-5. Bone marrow cells from WT, non-transgenic donor mice (CD45.1/CD45.2) were transplanted into lethally irradiated about 6-week-old Tie-2-eGFP-CLN-5 host mice (CD45.2) via retro-orbital injection. **a** At 10 weeks post-transplant, tail bleeds were performed to assess the efficacy of leukocyte substitution. FACS shows PBLs from non-irradiated, host eGFP-CLN-5 mice are all CD45.2^+^ (*left*), while those in chimeras are approx. 98% CD45.1^+^ CD45.2^+^ (*right*), indicating host PBLs were nearly entirely replaced. **b** Two days following confirmation of leukocyte substitution, EAE was induced in chimeras and age-matched non-irradiated, host eGFP-CLN-5 mice (at approx. 16 weeks of age; *n* = 4). At D8 EAE, PBLs were isolated from both groups of mice and percentages of eGFP-CLN-5^+^ CD45^+^ determined. Control PBLs from naïve, WT mice and PBLs labeled with isotype control antibodies were used to set the gates (data not shown). **c** z-stack confocal image from the spinal cord section of chimeric mouse at D9 EAE showing eGFP-CLN-5 (*green*) distribution along the intercellular boundaries of a CNS microvessel (*arrows*) and associated with an aggregate of perivascular leukocytes (*arrowhead*). *Insert* shows high-power field of aggregated eGFP-CLN-5^+^ leukocytes


To discount the possibility that the eGFP signal on leukocytes arose solely from endogenous expression of eGFP-CLN-5, due to some Tie-1 promoter activity in developing hemopoietic cells [73–75], analysis was repeated in bone marrow chimeras generated from non-transgenic donor (CD45.1/45.2) and lethally irradiated Tie-2-eGFP-CLN-5 recipient (CD45.2) mice. As shown in Fig. 4a, approximately 98% of circulating T cells in the chimeras was found to be CD45.1^+^ CD45.2^+^, indicating successful substitution of the leukocyte population of recipient Tie-2-eGFP-CLN-5 mice with that of donor mice. As leukocytes of chimeric mice are virtually exclusively donor-derived and, hence, lack the ability to endogenously express Tie-2 promoter-driven eGFP-CLN-5, the appearance of eGFP-CLN-5^+^ leukocytes in chimeras would reinforce the argument that the eGFP signal from these cells is endothelial in origin. Figure 4b, c and Additional file 8: Video 2 show that, as with Tie-2-eGFP-CLN-5 mice, chimeras displayed eGFP-CLN-5^+^ leukocytes within both the blood and CNS following EAE induction. The eGFP-CLN-5^+^ leukocytes in the CNS of chimeric mice again comprised a minor fraction of the extravasated cells and were further observed to aggregate, perhaps reflecting the ability of their CLN-5 to self-associate. Collectively, these findings suggest that a select population of leukocytes invading the CNS during neuroinflammation acquires CLN-5 in part from endothelial cells, and this acquisition occurs in blood.

Additional file 8Video 2. Aggregate formation of perivascular eGFP-CLN-5^+^ leukocytes. Video of z-stack confocal image from the spinal cord section of chimeric mouse at D9 EAE, showing an aggregate of perivascular eGFP-CLN-5^+^ leukocytes (green) and their multi-nucleated pattern with DRAQ5 (blue), adjacent to the same CNS microvessel in Fig. 4c.


### Expression of CLN-5 by endothelial-derived EVs

**Fig. 5:**
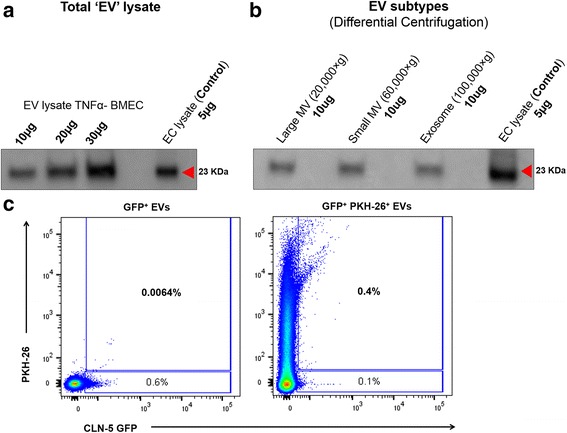
CLN-5^+^ expression in endothelial-derived EVs. **a**, **b** Western blot analysis of CLN-5 in EVs from supernatants of TNF-α-treated BMEC cultures. Total EVs (**a**) and exosome- and microvesicle-size EVs separated by differential ultracentrifugation (**b**), showing a 23 kDa molecular weight band consistent with the molecular weight of CLN-5. **c** FACS analysis of total EVs isolated from blood plasma of Tie-2-eGFP-CLN-5 mice with EAE, showing eGFP-CLN-5^+^ EVs alone (*left*) or eGFP-CLN-5^+^ EVs co-stained with PKH-26 dye to label all (eGFP-CLN-5^+^ and eGFP-CLN-5^−^) EVs (*right*), in the double-positive event gate. Approx. 0.4% of all blood-derived EVs are shown as eGFP-CLN-5^+^ PKH-26^+^. Nano-fluorescent-size standard beads (not shown) were used for setting the gates

To next examine if endothelial-derived EVs might act as potential sources of CLN-5 for leukocytes, expression of this protein was evaluated in isolated EV populations. First, “total” EVs (combined exosome- and microvesicle-sized-EVs) were isolated by differential centrifugation from the supernatants of cultured BMEC stimulated with TNF-α, a pro-inflammatory cytokine associated with EAE [63, 64] and a known inducer of endothelial EV release [44, 65]. The total EV pellet, as well as a lysate of the endothelial monolayer culture, expressed CLN-5 protein, as detected by Western blotting (Fig. 5a). TNF-α appeared to only modestly increase the amount of detectable CLN-5^+^ EVs released by BMEC (Additional file 9: Figure S7), indicating these are constitutively shed from these cells in culture. Further fractionating the EVs into exosomes and microvesicle-size vesicles, respectively, showed EV subtypes of both sizes expressed CLN-5 (Fig. 5b). FACS analysis of total EVs isolated from plasma of Tie-2-eGFP-CLN-5 mice with EAE additionally showed a fraction of these vesicles (0.4%) to be eGFP-CLN-5^+^ (Fig. 5c), suggesting their possible derivation from endothelial cells in vivo.

### Binding of endothelial-derived EVs to leukocytes

**Fig. 6:**
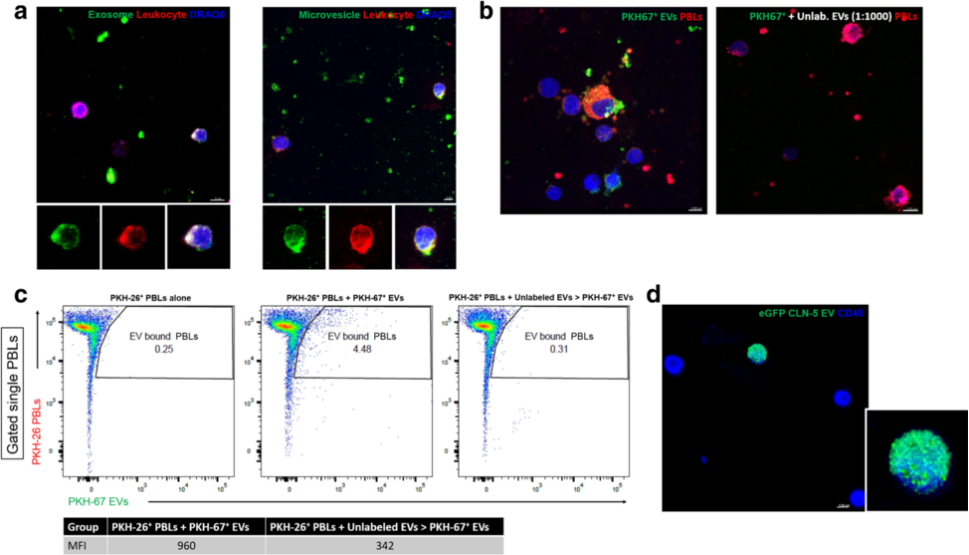
EVs from endothelial cells bind to leukocytes in vitro. **a**–**c** EVs collected from supernatants of TNF-α-treated BMEC cultures were incubated with isolated PBLs. **a** z-stack confocal images show PKH-67-labeled (*green*) exosome- and microvesicle-size EVs separated by differential ultracentrifugation can bind to PKH-26-labeled naïve PBLs (*red*), along with DRAQ5 staining of PBL nuclei (*blue*). *Insets* highlight co-localization of staining on a single PBL. **b**–**c** Binding of total PKH-67-labeled EVs to naïve PBLs. FACS analysis revealed 4.5% of the PBLs were PKH-26^+^ PKH-67^+^ (double-positive), suggestive of binding. The percentage of PBLs binding to PKH-67^+^ EVs diminished by 93%, while the PKH-67 signal intensity per leukocyte (mean fluorescence intensity), a reflection of the relative EV binding/cell, decreased by 65% in the presence of a 500-fold excess of unlabeled EVs. **d** Total eGFP-CLN-5^+^ EVs, FACS-purified from blood plasma of Tie-2-eGFP-CLN-5 mice at D8 EAE, bind to naïve PBLs in vitro. In this case, PBLs are identified by CD45 staining (*blue*). *Inset* further highlights a punctate binding pattern of eGFP-CLN-5^+^ EVs to PBLs

Having demonstrated endothelial-derived CLN-5 on both leukocytes and EVs, we next addressed whether EVs, in general, could bind to leukocytes—a fundamental action if EVs serve as physiological vehicles for endothelial-to-leukocyte transfer of CLN-5. EVs were again isolated from the cultured supernatant of TNF-α-stimulated BMEC and separated by differential centrifugation into exosome- and microvesicle-size fractions. The separate EV fractions were labeled with PKH-67 dye and incubated with PBLs obtained from naïve mice and labeled with PKH-26 dye. Evaluation by image analysis revealed both EV populations bound to leukocytes (Fig. 6a). FACS analysis confirmed this binding, as reflected by double-positive events, and further indicated a range of 4.5–7% of leukocytes bound to EVs in vitro (Fig. 6b). The binding of total PKH-67-labeled EVs to PKH-26-labeled leukocytes also appeared saturable (Fig. 6c), as it was sensitive to competition by excess of unlabeled EVs. Specifically, the percentage of leukocytes binding labeled EVs diminished ~93%, while the PKH-67 signal intensity per leukocyte (mean fluorescence intensity), a reflection of the relative EV binding/cell, fell ~65%.

To further explore if CLN-5^+^ EVs, in particular, could bind to leukocytes, total EVs (exosome- and microvesicle-size) were first isolated from plasma of Tie-2-eGFP-CLN-5 mice at D8 EAE and then eGFP-CLN-5^+^ EVs FACS-purified from this population for EV:leukocyte binding. As was the case with PKH-labeled EVs derived from BMEC, eGFP-CLN-5^+^ EVs obtained from plasma were observed to bind to leukocytes (Fig. 6d). Since these EVs could theoretically have been released into the circulation from any endothelial cells in the body, eGFP-CLN-5^+^ EVs were additionally isolated from culture supernatants of TNF-α-stimulated BMEC derived from Tie-2-eGFP-CLN-5 mice. EVs from this source, too, bound to leukocytes in vitro (data not shown), supporting the concept endothelial cells of the BBB, specifically, can shed CLN-5^+^ EVs capable of attaching to leukocytes.

### “EV-like” structures at the BBB endothelium and site of leukocyte attachment in situ

**Fig. 7:**
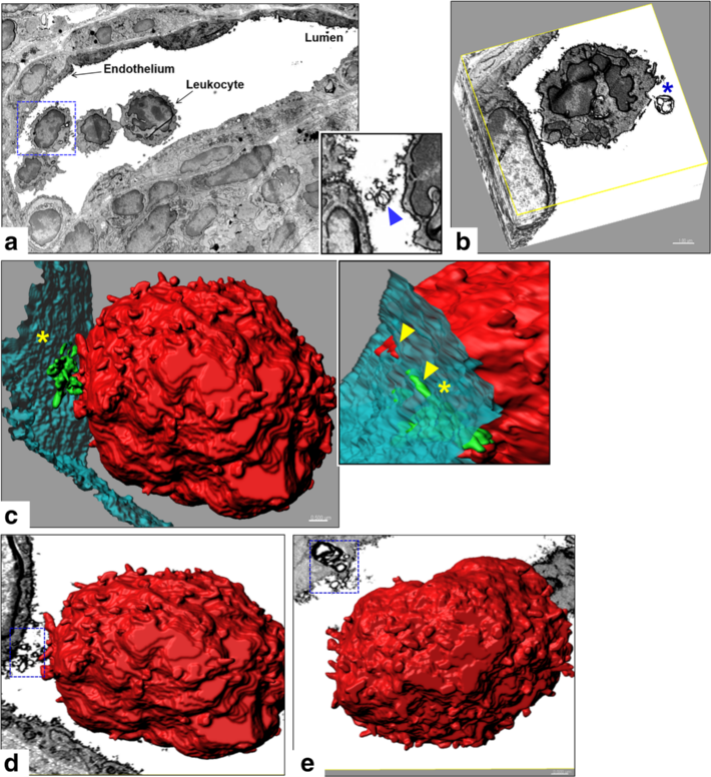
EV-like structures in situ at sites proximal to leukocyte adhesion. Serial EM images from mouse spinal cord sections obtained at D13 EAE. **a** Single serial section showing cross section of an inflamed venule highlighting adherent leukocytes, some apparently undergoing TEM. *Inset* highlights EV-like membrane-bound structures (*blue arrowhead*) at the leukocyte-endothelial interface of an adherent leukocyte (*blue box*). **b** Representative 3D reconstruction of an adherent leukocyte shown in **a**, generated from 130 serial slices. The larger membranous structure indicated by * is on the order of 1 μm in diameter and may represent an apoptotic body loosely tethered to the leukocyte. **c** Contour surface reconstruction of the “traced” leukocyte (*red*), EV-like structures (*green*), and endothelium (*turquoise*) in all 130 serial slices, providing a 3D view of all three elements at the site of leukocyte docking (*yellow asterisk*). *Inset* provides an oblique view of the site of leukocyte attachment, showing EV-like structures and leukocyte process (*yellow arrowheads*) apparently below the surface of the endothelial plasma membrane. **d**, **e** 3D surface reconstruction of the leukocyte in panels **a**–**c**, along with a representative serial EM slice, highlighting various patterns and sizes of EV-like structures, close to the site of leukocyte binding


Serial EM combined with 3D rendering was next used in an effort to try and capture EVs in association with leukocytes at the BBB in situ. Figure 7a, b shows an inflamed venule containing marginating leukocytes at various stages of extravasation, along with what appear as “EV-like” structures, ranging in size from ~100 nm to <1 μm, located between the leukocytes and the endothelium. These EV-like structures can also be seen in close approximation to the endothelial glycocalyx, as well as just subjacent to the endothelial plasma membrane (Fig. 7e). A 3D contour surface creation of a group of EV-like structures (Fig. 7c–e) depicts them as an aggregate seemingly partially enveloped—in an apparent “umbrella-like” manner—by a closely associated leukocyte (Fig. 7d). An oblique view of the rendered site of leukocyte:endothelial attachment further portrays the aggregate of EV-like structures as penetrating the endothelial surface, possibly reflecting a close continuity with nascent EVs (Fig. 7c; Additional file 10: Video 3).

Additional file 10Video 3. Endothelial EVs are released in situ at sites proximal to leukocyte adhesion. 3D surface reconstruction of an adherent leukocyte in the lumen of an inflamed microvessel in serial EM images from thoraco-lumbar spinal cord sections of a D13 EAE mouse. The contour surfaces were obtained by “tracing” the leukocyte (red), EVs (green), and endothelium (turquoise) in 130 serial sections using Imaris^®^, providing a 3D view of EV-like structures, apparently aggregated, proximal to the site of leukocyte attachment.


## Discussion

**Fig. 8:**
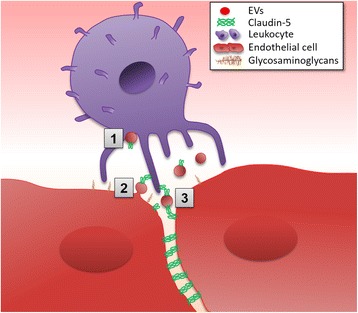
Interactions of endothelial CLN-5^+^ EVs with leukocytes. EVs shed from endothelial cells could potentially transfer CLN-5 protein to leukocytes and foster TEM by several conceivable scenarios: [1] binding of shed CLN-5^+^ EVs to undefined sites on the leukocyte surface, [2] binding of nascent CLN-5^+^ EVs still associated with the endothelium to endogenous CLN-5 on the leukocyte surface, and [3] binding of shed CLN-5^+^ EVs to endogenous CLN-5 on the leukocyte surface, resulting in temporary cross-linking of leukocyte to the endothelium. Binding of CLN-5^+^ EVs to endogenous CLN-5 on the leukocyte surface could potentially amplify leukocyte:endothelial interactions by increasing avidity of CLN-5-binding partners. Not shown are possibilities EVs might inject endothelial-derived CLN-5 protein and/or mRNA into the leukocyte for surface expression. Concentrated release of EVs near the junctional region could act in a juxtacrine manner to guide leukocytes to this site for TEM

This study established the presence of CLN-5^+^ leukocytes in the CNS during neuroinflammation and identified endothelial cells to be a source for this ectopic expression of CLN-5. Specifically, leukocytes bearing CLN-5 protein were observed in the blood and spinal cord of WT mice with EAE. The occurrence of eGFP-CLN-5^+^ leukocytes in the blood and CNS of Tie-2-eGFP-CLN-5 mice suggested the leukocyte eGFP signal can originate in part from endothelial cells. Endothelial contribution of eGFP was confirmed in chimeric mice, in which the endogenous leukocyte population of Tie-2-eGFP-CLN-5 mice was replaced with bone marrow-derived cells from non-transgenic mice, so as to preclude any transgenic Tie-2 activity in the leukocyte population of the recipients. The appearance of eGFP-CLN-5^+^ leukocytes in these chimeric mice is consistent with a transfer of eGFP-CLN-5 from endothelial cells to circulating leukocytes. Studies additionally demonstrated circulating EVs from mice with EAE, as well as EVs released from TNF-α-stimulated cultured BMEC, contained CLN-5 and could bind to leukocytes in vitro. In situ analysis by serial electron microscopy further revealed what appear to be EV-like structures enveloped by leukocytes marginating along the CNS microvascular endothelium. Collectively, these findings allude to a possible scenario during neuroinflammation, wherein endothelial cells at the BBB release CLN-5^+^ EVs that subsequently bind to leukocytes in a juxtacrine manner (Fig. 8).

The present work extends the report of Mandel et al. [20, 21], which noted an increase in CLN-5^+^ leukocytes in blood from MS patients who experienced disease relapse. Our findings corroborated the presence of CLN-5^+^ leukocytes in blood during EAE, an animal model of MS [54–56], and further demonstrated their appearance in the CNS. The presence of these cells in both blood and CNS relatively early during disease (D9 EAE) suggests it unlikely they acquire CLN-5 as a consequence of extravasation or only when there is substantial BBB breakdown later in the chronic phase [58, 67]. Specifically, these findings argue against CLN-5^+^ leukocytes arising merely as a result of having passively picked up CLN-5 remnants of proteolyzed endothelial TJs that may have been deposited abluminally [76]. An alternative scenario is leukocytes acquire CLN-5 from endothelial cells while in the circulation as a prelude to extravasation. Anecdotal support for this endothelial-to-leukocyte transfer hypothesis is further provided by observations of Mandel et al. [20] who noted that, while levels of CLN-5 were elevated in PBLs of MS patients in the relapse state, CLN-5 status was unaffected by in vitro activation of these cells. The authors concluded that upregulation of CLN-5 by leukocytes in MS reflects interaction of these cells with “additional cell types.” That expression of CLN-5 by PBLs from MS patients was diminished following successful anti-inflammatory therapy is also consistent with the argument that acquisition of CLN-5 by leukocytes is not merely a result of leukocyte extravasation but, rather, occurs prior to it—while the cells are still in the blood.

Acquisition of CLN-5 by leukocytes in the blood would also be in line with endothelial-to-leukocyte transfer of this protein being mediated by EVs. That both total EVs as well as CLN-5^+^ EVs bound to leukocytes in vitro further underscores the feasibility of such a transaction. Also, lending support are reports by Takahashi et al. [40] and Haqqani et al [39] that cultures of human aortic endothelial cells and human BMEC, respectively, release EVs containing junctional proteins. Most recently, Andrews et al. [41] described the release from cultured human BMECs of EVs containing the TJ protein occludin, following application of mechanical trauma to these cells, and additionally noted EVs bearing this protein were elevated in blood plasma of mice subject to traumatic brain injury (TBI). Further paralleling our results for CLN-5^+^ vesicles, these authors observed that occludin^+^ EVs stemming from trauma were of both exosome and microvesicle size. As mechanical trauma to the CNS microvasculature is also associated with endothelial activation, immune reaction, and neuroinflammation [77–79], BMEC release of EVs carrying TJ proteins might be a general phenomenon linked to CNS leukocyte infiltration. Notably, Andrews et al. [41] also found the percent of occludin^+^ EVs in the blood of TBI mice to be comparatively minor, e.g., <6% of total EVs, compared to ~1% of CLN-5^+^ EVs detected in mice with early-stage EAE. The slight discrepancies in the observed percentages could be due to differences in the experimental conditions employed, with TBI possibly being more disruptive to the vasculature. Also, FACS detection of EVs by forward scatter properties [41] versus side scatter properties (this study) could have been a contributing factor as well. The relatively low level of CLN-5^+^ EVs detected in the circulation is also in accord with their being released focally from select endothelial cells, rather than diffusely as part of a widespread endothelial response.

It is further significant that only a fraction of leukocytes (range 4.5–7%) was observed to bind to EVs in vitro, as this qualitatively resembled the similarly limited populations of CLN-5-immunostained and eGFP-CLN-5^+^ leukocytes observed in the CNS of EAE mice. This correlation lends added credence to EVs being a major mode by which leukocytes acquire CLN-5 and might further imply only a specialized subset of leukocytes expresses or utilizes binding sites for EVs. As binding of labeled EVs was also sensitive to competition by an excess of unlabeled EVs, binding sites for EVs on leukocytes appear to be saturable. The identity of these sites is not yet clear but might include heparan sulfate proteoglycans, which are abundant on leukocytes and have been shown to bind to EVs in a manner inhibited by free heparan sulfate chains [80].

The binding to leukocytes of CLN-5^+^ EVs, in particular, could potentially occur through another mechanism. Our finding that leukocytes from naïve mice and those with EAE express CLN-5 mRNA is in accord with a previous observation of CLN-5 gene expression by circulating leukocytes from both healthy controls and MS patients [20]. In turn, de novo expression of CLN-5 or other TJ proteins by leukocytes might provide binding sites for endothelial-derived CLN-5^+^ EVs. Even if only minimal TJ protein is initially expressed on the leukocyte cell surface, this could be sufficient for binding CLN-5^+^ EVs in a homophilic and/or heterophilic manner. A modest elevation of endogenous CLN-5 expression by leukocytes during neuroinflammation, as was seen here during EAE and in MS patients during disease exacerbation [20], might further stimulate binding. Such EV binding might allow for an acute and significant increase in CLN-5 on the leukocyte surface in response to local cues, such as endothelial activation and stimulated EV release at restricted sites. Acquiring most of their CLN-5 in a temporally and spatially restricted way would avert a need for leukocytes to endogenously express significant amounts of CLN-5 protein and the risk of extensively binding each other and forming aggregates in the general circulation, as do other cell types engineered to ectopically express CLNs [81]. It is significant in this regard that although circulating leukocytes from naïve mice display CLN-5 gene expression, no CLN-5^+^ leukocytes—or any perivascular infiltrates—were detected in the CNS of these animals. Apparently, CLN-5 gene expression by leukocytes is not sufficient to drive these cells’ entry into the CNS in the absence of other cues. It is only within the context of neuroinflammation, and possibly the focal release and binding of endothelial-derived CLN-5^+^ EVs, that CLN-5^+^ leukocytes make their detectable CNS appearance.

While the functional significance of leukocytes displaying CLN-5 awaits clarification, appearance of these cells in both blood and CNS satisfies a minimal criterion for a zipper mechanism of TEM [15–18] to potentially operate in the CNS. A priori, a scenario wherein CLN-5 is focally transferred from endothelial cells to marginating leukocytes at the inflamed BBB, for the purpose of promoting TEM through transient homophilic and/or heterophilic interactions between CLN partners on the two cell types, is in accord with several lines of evidence. Notable in this regard is that leukocyte adhesion to the vascular wall is a stimulus for EV release [52], providing for a tight coupling of leukocyte margination and EV release in space and time. Indeed, with the capability of CLN-5^+^ EVs to act as multidentate ligands, their binding to CLN-5 on leukocytes might allow for the type of cross bridging depicted in Fig. 8, supporting enhanced opportunity for leukocyte and endothelial TJ protein partners to engage through a zipper mechanism. This scheme would endorse a juxtacrine interaction between leukocytes at the luminal surface and nascent EVs and account for the relatively low frequency of CLN-5^+^ leukocytes and CLN-5^+^ EVs in the general circulation. Serial SEM observations of EV-like structures in situ interfaced between leukocytes and endothelial cells at the BBB comport with such a juxtacrine interaction. Lending added credence to this hypothesis is the finding by Jimenez at al. [44] that complexes of monocytes with EVs derived from TNF-α-stimulated cultured human BMECs facilitated TEM across an in vitro model of the BBB. The additional observation that CLN-5^+^ leukocytes in the CNS tend to remain in close proximity to vessels and not enter deep into the parenchyma might further argue the action of these cells is brief and largely confined to the extravasation process itself. Making use of a zipper mechanism, CLN-5^+^ leukocytes might serve the role of “pioneers” to open the BBB for other leukocytes to closely follow through partially patent inter-endothelial junctions. Such a pioneering function would also be consistent with the modest numbers of these cells found in the blood and CNS. Characterization of CLN-5^+^ leukocyte function is beyond the scope of this study and is currently being pursued by this laboratory at several levels.

New roles for EVs in the CNS are continuing to emerge [82], with EV-mediated RNA transfer from immune cells to neurons [75] and from glioblastoma to microglia/macrophages [83], both being demonstrated in the brain. EVs derived from endothelial cells of the BBB, in particular, have further garnered attention for their roles in neurodegenerative disease [84] and communication with other cell types in the neurovascular unit [85]. It may further be that reports of increased circulating TJ proteins following brain trauma [86] and hemorrhage [87] actually reflect release into the bloodstream of TJ protein^+^ EVs from an inflamed and/or damaged BBB. Indeed, the propensity for EVs derived from inflamed BMEC to self-aggregate [85] might be a manifestation of TJ proteins on the EV surface. The present studies offer a novel rationale for the increasing association of EVs with neuroinflammation [88–93]. Specifically, endothelial-to-leukocyte transfer of CLN-5 and other TJ proteins via EVs could be a basis for the ectopic display by leukocytes of junctional components normally restricted to specialized inter-endothelial/epithelial contacts and potentially allow for a zipper mechanism for leukocyte extravasation across the BBB. In this respect, CLN-5^+^ EVs released from BMEC might “educate” leukocytes in migratory behavior, paralleling what EVs from tumor cells have been shown to do in stimulating mobilization of bone marrow progenitor cells out of their niche [94, 95]. As more factors are identified that foster paracellular versus transcellular TEM [94] across the BBB, EVs released by CNS endothelial cells might be found to serve as additional determinants. Interestingly, the recent observation of elevated circulating TJ proteins associated with leukemia central nervous system metastasis [96] may indicate tumor cells and leukocytes share a common EV-mediated mechanism to cross the BBB and other tight barriers. Further analysis of EV action during neuroinflammation will aid in illuminating pathogenic mechanisms and potential biomarkers of disease status, as well as offer new therapeutic prospects to antagonize EVs or exploit their properties for molecular delivery [97–101].

## Conclusions

In summary, this study provides the first evidence of CLN-5^+^ leukocytes within the CNS during the neuroinflammatory condition EAE. CLN-5^+^ leukocytes were also observed in blood, paralleling findings described in MS patients [20]. Endothelial cells were further determined to be at least one source of this ectopic expression of CLN-5. EVs bearing CLN-5 protein, either released into the circulation during EAE or into the media of TNF-α-stimulated BMEC cultures, bound to leukocytes in vitro, suggesting EVs could act as vehicles for endothelial-to-leukocyte transfer of CLN-5 protein at the BBB. Serial EM combined with 3D rendering additionally showed EV-like structures within the lumen of inflamed CNS microvessels, positioned between marginating leukocytes and endothelial cells. Collectively, these findings could provide a basis for a zipper mechanism to facilitate TEM into the CNS through the formation of temporary TJ protein bridges between leukocytes and endothelial cells at the BBB.

## Additional files

Additional file 1: Figure S1. Cultured BMEC from Tie-2-eGFP-CLN-5 mice. z-stack confocal image of primary BMEC culture from Tie-2-eGFP-CLN-5 mice, revealing TJ protein CLN-5 (green) at the inter-endothelial regions and DRAQ5-stained nuclei (blue). (TIF 1.47 mb)
Additional file 2: Figure S2. Nanoparticle tracking analysis of EVs. A NS300 NTA was used to perform high-resolution particle-size profiling and concentration measurements of exosome- (left) and microvesicle-size particles (right) purified by differential centrifugation from the supernatant of TNF-α-stimulated mouse BMEC culture. (TIF 1.28 mb)
Additional file 3: Figure S3. Gating strategy. (a–c) Gating for PBLs. PBLs (circulating leukocytes) isolated from naïve WT mice were gated on a forward scatter/side scatter (FSC-A/SSC-A) dot plot (a). These events were next visualized using a FSC-W/FSC-H dot plot, and the singlets (single cells) were gated (b). Following PKH-26 dye (red) labeling, single PBLs were displayed on a PKH-26/PKH-67 dot plot (c). (d) Gating for EVs. For detecting EVs, nano-fluorescent beads were first used to determine the limit of detection of submicron particles using both side scatter and green fluorescence detectors and to gate on events that lay outside of the background. EVs labeled with PKH-67 (green) alone were used to set gates for free/unbound EVs and shown on a PKH-26/PKH-67 dot plot. (TIF 1.24 mb)
Additional file 5: Figure S4. CLN-5^+^ leukocytes along the meningeal and parenchymal thoraco-lumbar spinal cord venules in EAE. z-stack confocal images acquired from a thoraco-lumbar spinal cord cryosection of WT mice at D9 EAE are shown, revealing staining for TJ protein CLN-5 (green), endothelial CD31 (red), and nuclear DRAQ5. Regions A and B are shown to highlight the emergence of CLN-5^+^ leukocytes along the meningeal microvessels, whereas C underscores the TEM of CNS-infiltrating CLN-5^+^ leukocytes in parenchymal microvessels during EAE progression. (TIF 1.68 mb)
Additional file 6: Figure S5. Co-localization of CLN-5 and CD45. Representative z-stack confocal images show double staining of isolated, fixed PBLs with simultaneously applied CLN-5 (green) and CD45 (blue) antibodies. Some steric hindrance may exist between the two antibodies, as PBLs that bind higher CD45 antibody have a corresponding reduced CLN-5 signal. (TIF 662 kb)
Additional file 7: Figure S6. CLN-5 mRNA in PBLs. PBLs were isolated from naïve and immunized mice at D8 and D13 post-EAE induction. Relative CLN-5 mRNA expression values in isolated PBLs determined by qRT-PCR are shown. mRNA values are presented as mean percent expression relative to RPL-19 (Mean ± SEM). One-way ANOVA, followed by Tukey’s multiple comparison post hoc analysis, was used to compare the three groups. **p* < 0.05, *n* = 4. (TIF 198 kb)
Additional file 9: Figure S7. TNF-α treatment of BMECs and CLN-5^+^ EV production. Total EVs were obtained by differential ultracentrifugation from untreated control or TNF-α-treated BMECs and analyzed by Western blotting. Only a moderate increase in the intensity of the 23-kDa CLN-5 band was observed in EVs from TNF-α-treated BMECs. BMEC cell lysate or lysate from isolated mouse brain microvessels were used as positive controls on the same blot. LI-COR Image Studio v5.2 was used to perform densitometric analysis. Relative band intensities above the background were recorded. (TIF 317 kb)

## Abbreviations

BBB: Blood-brain barrier
BMEC: Brain microvascular endothelial cells
CLN: Claudin
CNS: Central nervous system
EAE: Experimental autoimmune encephalomyelitis
EVs: Extracellular vesicles
TJ: Tight junction
