# Supplementary figures and images for: Appearance of claudin-5+ leukocytes in the central nervous system during neuroinflammation: a novel role for endothelial-derived extracellular vesicles
Source: J Neuroinflammation. 2016 Nov 16;13:292. doi: 10.1186/s12974-016-0755-8 (PMC5112695; doi:10.1186/s12974-016-0755-8)

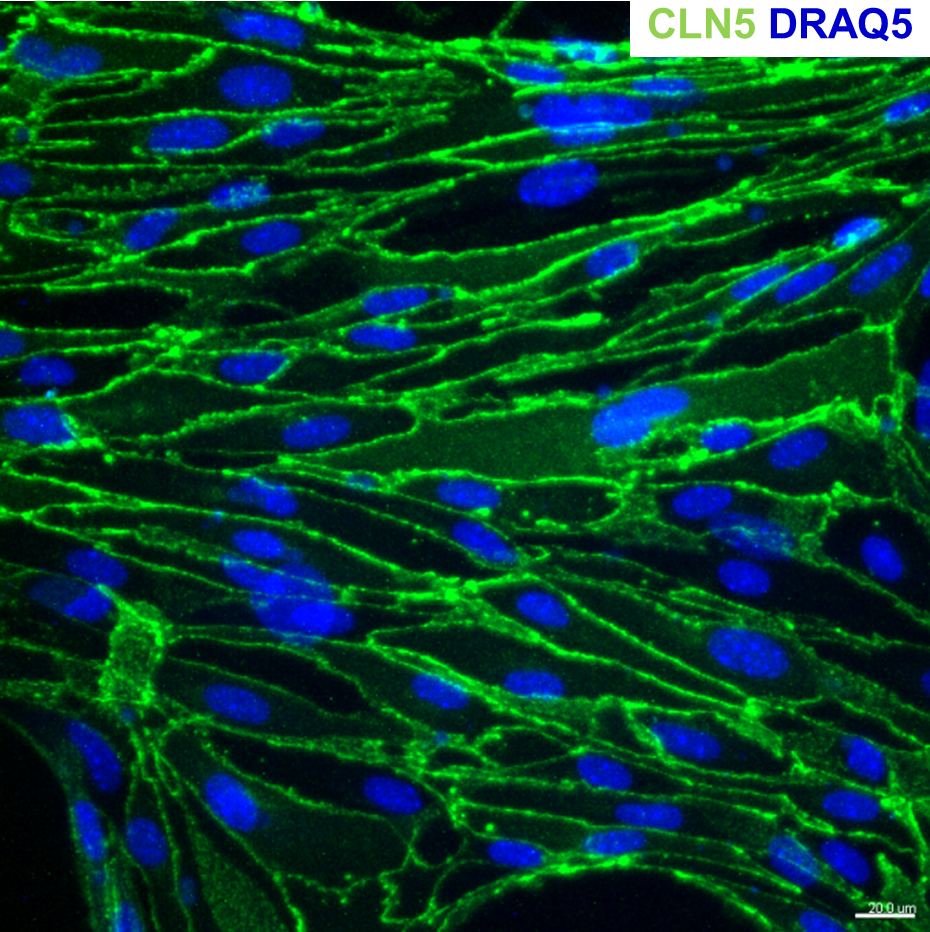

Supplement: Additional file 1: Figure S1. — Cultured BMEC from Tie-2-eGFP-CLN-5 mice. z-stack confocal image of primary BMEC culture from Tie-2-eGFP-CLN-5 mice, revealing TJ protein CLN-5 (green) at the inter-endothelial regions and DRAQ5-stained nuclei (blue). (TIF 1.47 mb) [file 12974_2016_755_MOESM1_ESM.tif]

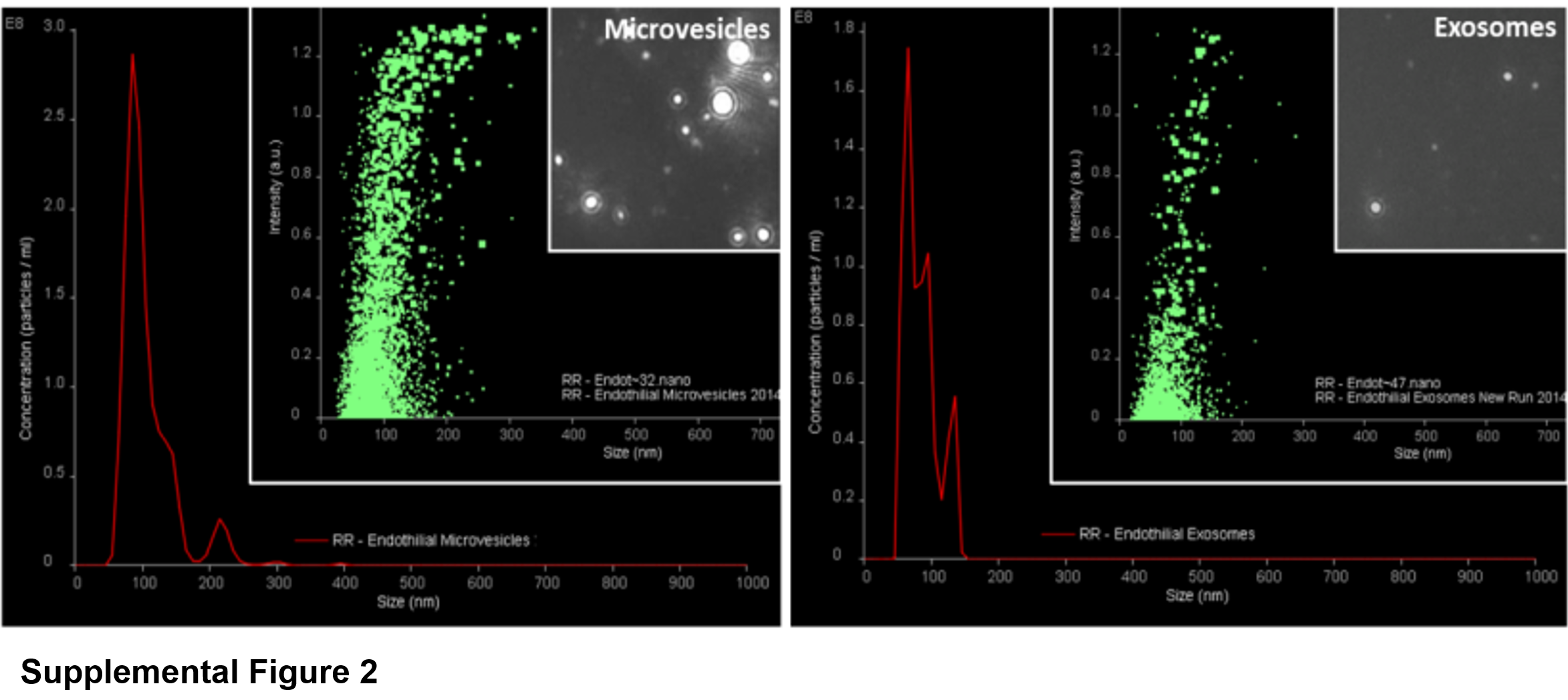

Supplement: Additional file 2: Figure S2. — Nanoparticle tracking analysis of EVs. A NS300 NTA was used to perform high-resolution particle-size profiling and concentration measurements of exosome- (left) and microvesicle-size particles (right) purified by differential centrifugation from the supernatant of TNF-α-stimulated mouse BMEC culture. (TIF 1.28 mb) [file 12974_2016_755_MOESM2_ESM.tif]

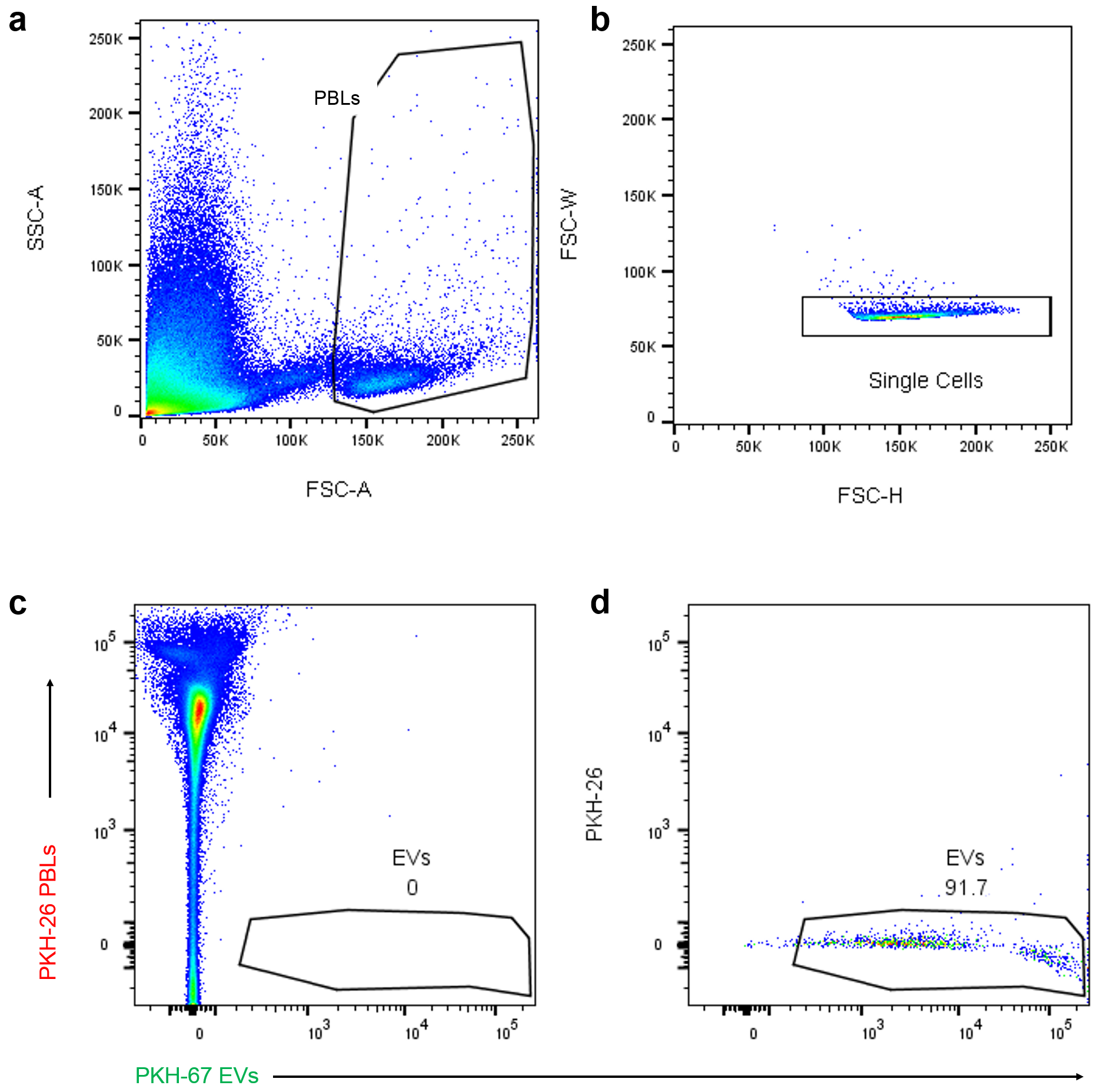

Supplement: Additional file 3: Figure S3. — Gating strategy. (a–c) Gating for PBLs. PBLs (circulating leukocytes) isolated from naïve WT mice were gated on a forward scatter/side scatter (FSC-A/SSC-A) dot plot (a). These events were next visualized using a FSC-W/FSC-H dot plot, and the singlets (single cells) were gated (b). Following PKH-26 dye (red) labeling, single PBLs were displayed on a PKH-26/PKH-67 dot plot (c). (d) Gating for EVs. For detecting EVs, nano-fluorescent beads were first used to determine the limit of detection of submicron particles using both side scatter and green fluorescence detectors and to gate on events that lay outside of the background. EVs labeled with PKH-67 (green) alone were used to set gates for free/unbound EVs and shown on a PKH-26/PKH-67 dot plot. (TIF 1.24 mb) [file 12974_2016_755_MOESM3_ESM.tif]

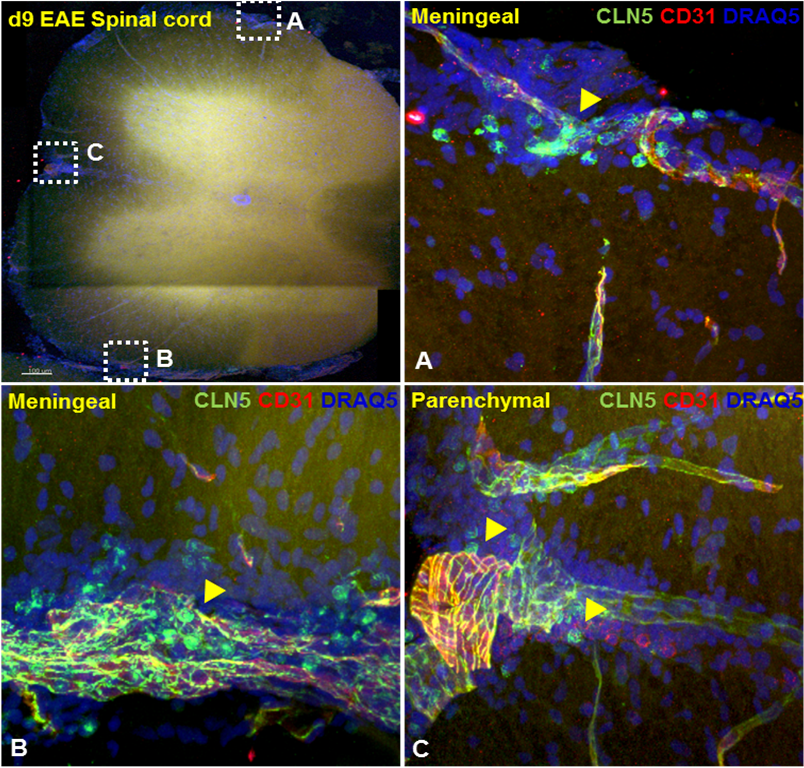

Supplement: Additional file 5: Figure S4. — CLN-5+ leukocytes along the meningeal and parenchymal thoraco-lumbar spinal cord venules in EAE. z-stack confocal images acquired from a thoraco-lumbar spinal cord cryosection of WT mice at D9 EAE are shown, revealing staining for TJ protein CLN-5 (green), endothelial CD31 (red), and nuclear DRAQ5. Regions A and B are shown to highlight the emergence of CLN-5+ leukocytes along the meningeal microvessels, whereas C underscores the TEM of CNS-infiltrating CLN-5+ leukocytes in parenchymal microvessels during EAE progression. (TIF 1.68 mb) [file 12974_2016_755_MOESM5_ESM.tif]

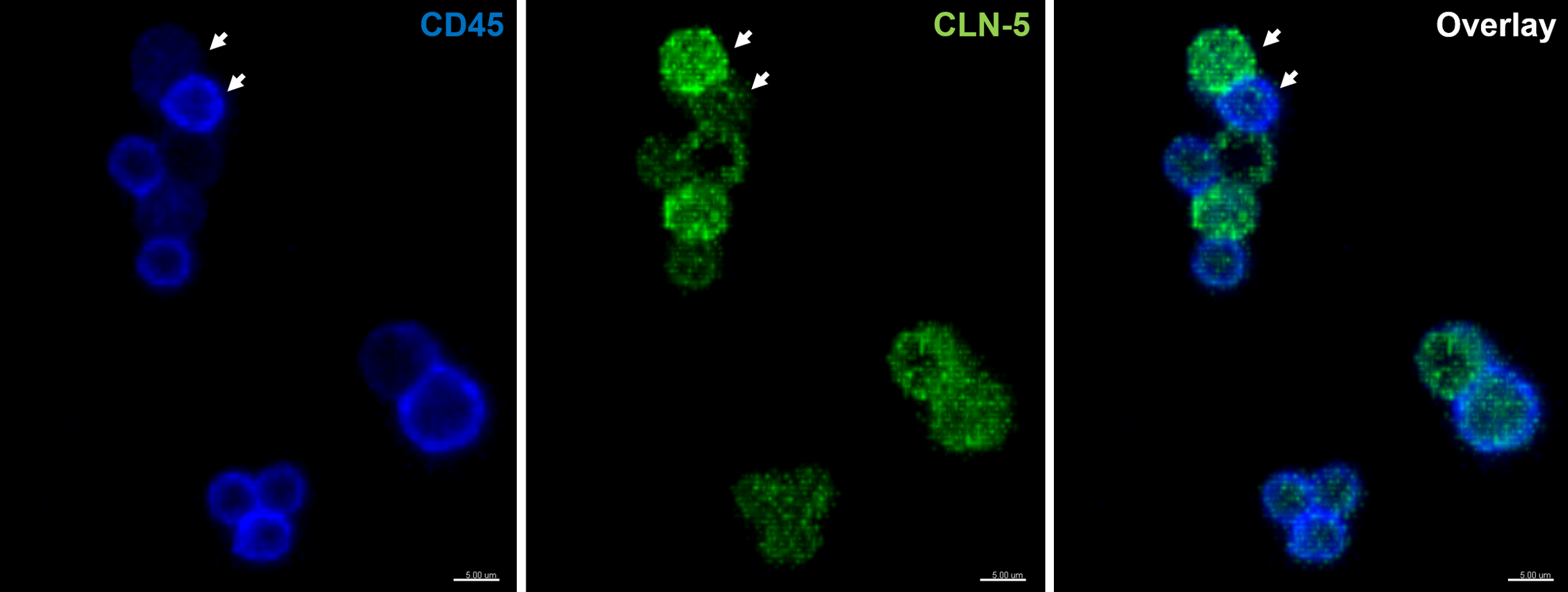

Supplement: Additional file 6: Figure S5. — Co-localization of CLN-5 and CD45. Representative z-stack confocal images show double staining of isolated, fixed PBLs with simultaneously applied CLN-5 (green) and CD45 (blue) antibodies. Some steric hindrance may exist between the two antibodies, as PBLs that bind higher CD45 antibody have a corresponding reduced CLN-5 signal. (TIF 662 kb) [file 12974_2016_755_MOESM6_ESM.tif]

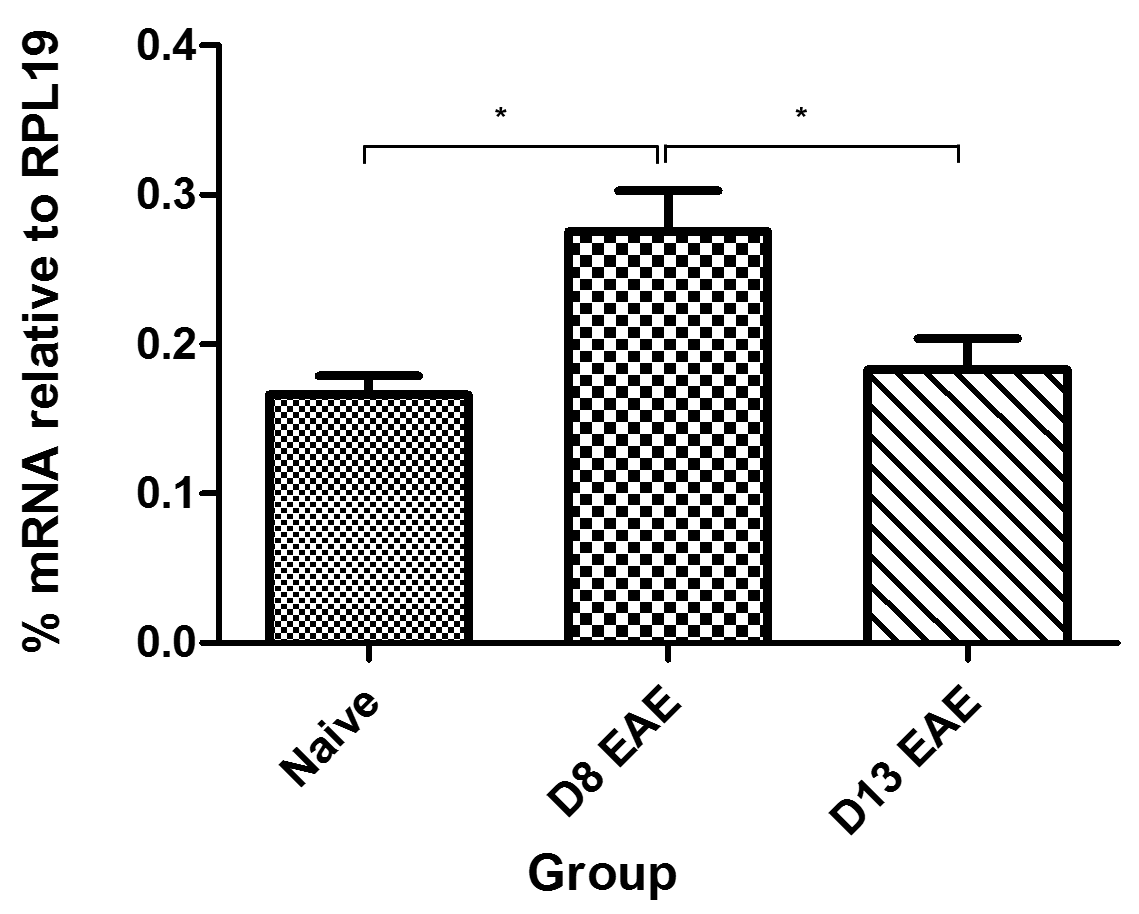

Supplement: Additional file 7: Figure S6. — CLN-5 mRNA in PBLs. PBLs were isolated from naïve and immunized mice at D8 and D13 post-EAE induction. Relative CLN-5 mRNA expression values in isolated PBLs determined by qRT-PCR are shown. mRNA values are presented as mean percent expression relative to RPL-19 (Mean ± SEM). One-way ANOVA, followed by Tukey’s multiple comparison post hoc analysis, was used to compare the three groups. *p < 0.05, n = 4. (TIF 198 kb) [file 12974_2016_755_MOESM7_ESM.tif]

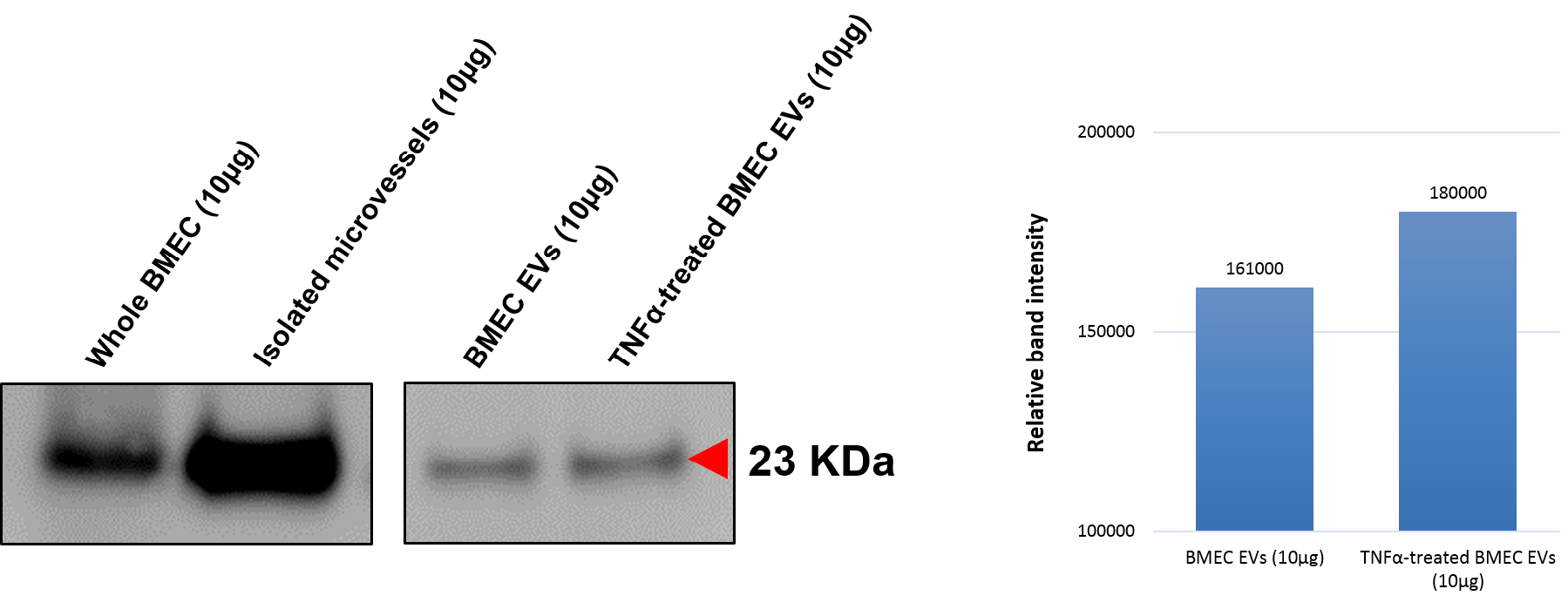

Supplement: Additional file 9: Figure S7. — TNF-α treatment of BMECs and CLN-5+ EV production. Total EVs were obtained by differential ultracentrifugation from untreated control or TNF-α-treated BMECs and analyzed by Western blotting. Only a moderate increase in the intensity of the 23-kDa CLN-5 band was observed in EVs from TNF-α-treated BMECs. BMEC cell lysate or lysate from isolated mouse brain microvessels were used as positive controls on the same blot. LI-COR Image Studio v5.2 was used to perform densitometric analysis. Relative band intensities above the background were recorded. (TIF 317 kb) [file 12974_2016_755_MOESM9_ESM.tif]
